# Long-Term MRI Cell Tracking after Intraventricular Delivery in a Patient with Global Cerebral Ischemia and Prospects for Magnetic Navigation of Stem Cells within the CSF

**DOI:** 10.1371/journal.pone.0097631

**Published:** 2014-06-11

**Authors:** Miroslaw Janowski, Piotr Walczak, Tomasz Kropiwnicki, Elzbieta Jurkiewicz, Krystyna Domanska-Janik, Jeff W. M. Bulte, Barbara Lukomska, Marcin Roszkowski

**Affiliations:** 1 NeuroRepair Department, Mossakowski Medical Research Centre, Polish Academy of Sciences, Warsaw, Poland; 2 Department of Neurosurgery, Mossakowski Medical Research Centre, Polish Academy of Sciences, Warsaw, Poland; 3 Russell H. Morgan Department of Radiology and Radiological Science, Division of Magnetic Resonance Research, The Johns Hopkins University School of Medicine, Baltimore, Maryland, United States of America; 4 Cellular Imaging Section and Vascular Biology Program, Institute for Cell Engineering, The Johns Hopkins University School of Medicine, Baltimore, Maryland, United States of America; 5 Department of Radiology, Faculty of Medical Sciences, University of Warmia and Mazury, Olsztyn, Poland; 6 Department of Neurosurgery, The Children’s Memorial Health Institute, Warsaw, Poland; 7 Department of Radiology, Magnetic Resonance Unit, The Children’s Memorial Health Institute, Warsaw, Poland; 8 Department of Chemical and Biomolecular Engineering, The Johns Hopkins University School of Medicine, Baltimore, Maryland, United States of America; 9 Department of Biomedical Engineering, The Johns Hopkins University School of Medicine, Baltimore, Maryland, United States of America; University of South Florida, United States of America

## Abstract

**Background:**

The purpose of the study was to evaluate the long-term clinical tracking of magnetically labeled stem cells after intracerebroventricular transplantation as well as to investigate *in vitro* feasibility for magnetic guidance of cell therapy within large fluid compartments.

**Method:**

After approval by our Institutional Review Board, an 18-month-old patient, diagnosed as being in a vegetative state due to global cerebral ischemia, underwent cell transplantation to the frontal horn of the lateral ventricle, with umbilical cord blood-derived stem cells labeled with superparamagnetic iron oxide (SPIO) contrast agent. The patient was followed over 33 months with clinical examinations and MRI. To evaluate the forces governing the distribution of cells within the fluid compartment of the ventricular system *in vivo*, a gravity-driven sedimentation assay and a magnetic field-driven cell attraction assay were developed *in vitro*.

**Results:**

Twenty-four hours post-transplantation, MR imaging (MRI) was able to detect hypointense cells in the occipital horn of the lateral ventricle. The signal gradually decreased over 4 months and became undetectable at 33 months. *In vitro,* no significant difference in cell sedimentation between SPIO-labeled and unlabeled cells was observed (p = NS). An external magnet was effective in attracting cells over distances comparable to the size of human lateral ventricles.

**Conclusions:**

MR imaging of SPIO-labeled cells allows monitoring of cells within lateral ventricles. While the initial biodistribution is governed by gravity-driven sedimentation, an external magnetic field may possibly be applied to further direct the distribution of labeled cells within large fluid compartments such as the ventricular system.

## Introduction

Stem and progenitor cell-based therapy is considered a new avenue for the treatment of various diseases for which there is no effective cure [1], [2]. Neurological diseases pose a special challenge due to the complexity of the central nervous system (CNS) [3], [4]. There have been a few reports on successful, open-label cell therapy trials for Parkinson’s disease, [5], [6]. However, double-blind trials failed to reveal a statistically significant improvement, which was in part due to the high variability of the obtained outcomes [7]–[9]. Nevertheless, cell transplantation experiments are being performed preclinically and clinically in dozens of otherwise untreatable neurological disorders [10]. Intraparenchymal stereotaxic injection has initially been the method of choice for targeting cells toward well-defined anatomical locations. Systemic (i.v.) injections have also been used in several clinical trials [11], [12]. A major obstacle in the evaluation of these clinical trials is the uncertainty if cells are delivered correctly at the desired location and/or reach their target successfully. For intracebroventricular (ICV) injections, non-invasive visualization of cells is of particular importance as the cell dispersion is dictated by cerebro-spinal fluid (CSF)-driven flow mechanisms where the distribution of injected cells can be highly variable.

MRI cell tracking has recently gained attention as a clinically applicable tool to track cells non-invasively in real-time [13]. These initial clinical studies, performed in patients with cancer [14], brain trauma [15], multiple sclerosis [16], and diabetes [17] have demonstrated proof of feasibility of clinical detection. The very rigorous study performed on healthy volunteers has just confirmed safety of cell labeling by super-paramagnetic iron oxide SPIO [18]. For these studies, the longest time frame for follow upis 6 months [16]. The early outcome in a severely, globally ischemic patient who was transplanted ICV with autologous cord-blood-derived, SPIO-labeled neural progenitors, was reported previously [19]. In this study, we present a long-term imaging evaluation where the patient was followed for 33 months. Since only 20 percent of transplanted cells were labeled in this clinical experiment, additional *in vitro* fluid-phase studies modeling the movements of SPIO-labeled and unlabeled cells were conducted to gain a better insight about the fate of transplanted cells *in vivo*. CSF circulation directs the flow from the lateral ventricle to the subarachnoid space within the posterior fossa; this flow can potentially affect the distribution of injected cells. SPIO-labeling and subsequent MRI visualization may be used to evaluate such a possible CSF-driven biodistribution. Since sedimentation resulting from the gravity can also be a leading factor determining cell distribution within a fluid compartment [20], we performed an *in vitro* assay to compare the speed of sedimentation of SPIO-labeled vs. non-labeled cells. We also demonstrate here the potential for guiding the ICV distribution of SPIO-labeled cells with the use of an external magnetic field.

## Materials and Methods

### 2.1 Patient History

A nine-month-old patient was in a vegetative state as a result of global cerebral ischemia. An extensive rehabilitation program over three months did not result in any recovery, and a permanent vegetative state was diagnosed [21]. MR imaging revealed a mild global atrophy without focal lesions. Experimental cell therapy was considered due to extremely poor prognosis. The patient’s own cord blood was deposited at birth in a private blood bank; the parents of the patient decided to store his cord blood and covered all expenses related to it. The access to patient’s own source of stem cells facilitated the decision on cell transplantation. The parents provided written informed consent to include the patient in the study and have potentially personally identifying information published. The clinical study was conducted in Warsaw after approval by the Institutional Review Board (Bioethics Committee) at the Children’s Memorial Health Institute, Warsaw, Poland.

Briefly, autologous cord blood nucleated cells obtained during full-time delivery (2.4×10^7^ cells/ml stored in 10% DMSO) were thawed and cultured for 10 days in previously described neurogenic conditions [22] in a GMP facility. A total of 3.6×10^7^ cells were delivered in three equal doses, with the injections performed at one-month intervals. For the first dose of 1.2×10^7^ cells, 20% of cells were labeled with Feridex/PLL as previously described [19]. The two other injections were given with unlabeled cells only. The transplantation procedure was performed under general anesthesia and 0.5 ml of cells in saline was delivered to the anterior horn of the right lateral ventricle using a standard shunt drain. The patient was operated in supine position, typical for shunt drain placement. MR images were obtained before and at 1 day, 1 week, 1 month, 2 months, and 4 months after the first injection using a 1.5 T scanner equipped with an 8-channel phased-array head coil (Magnetom Sonata Maestro Class, Siemens, Germany). For the detection of SPIO signal, a SWI imaging sequence was used: slabs = 1, phase R-L, slice oversampling = 11, slices per slab = 72, FOV 230 mm, slice thickness 1.6 mm, dist factor 20%, averages = 1, TR/TE = 49/40, flip angle = 15, matrix 168×256, voxel size 1.2×0.9×1.6 mm. A neurological examination was performed six months after the first transplantation. The patient was then admitted to the hospital for clinical and radiological evaluation up to 33 months after the first cell transplantation. Osirix (Pixmeo) and Amira (Visage Imaging) software were used for image processing. The entire procedure was performed primarily for therapeutic purposes and the use of labeled cells was only a side-aspect of the whole treatment.

### 2.2 Cell Sedimentation Assay

Non-transformed line of neural stem cells derived from human umbilical cord blood (HUCB-NSC) was used for *in vitro* study [23]. HUCB-NSC were cultured in DMEM/F12 supplemented with 2% fetal bovine serum, 1% insulin-transferrin-selenium, and 1% antibiotic/antimycotic solution (Invitrogen). Fifty percent of cells were labeled for two days with Molday ION Rhodamine B-conjugated iron oxide nanoparticles (25 µg/ml, MIRB, BioPAL) and the transfection agent poly-L-Lysine (Sigma P-1524, 375 ng/ml) [24]. The unlabeled fraction of cells was counterstained with CellTracker cGreen (Molecular Probes). For sedimentation experiments, both red (SPIO-labeled) and green (unlabeled) cells were mixed to reach a final working solution of 2,000 cells/µl.

Sedimentation chambers were designed by removing the convex tip of 10 mm NMR tubes (New Era Enterprises, INC). The cylinders were then placed vertically on round glass-bottom, 1.5-inch cell culture dishes (RD-1.5-UNC, LiveAssay), and were thermally glued to form a waterproof seal. The resulting sedimentation chambers had a height of 15 cm and a diameter of 1 cm. They were filled with 10 mM PBS and seeded with a small amount of Hoechst-labeled cells in order to determine the bottom level of the sedimentation chamber for focusing of the microscope. A Zeiss inverted microscope (Axiovert 200 M) was programmed to acquire both red and green channel images automatically at one-minute intervals. Ten µl of 50/50 labeled/unlabeled cell suspension (20,000 cells) was placed on top of the sedimentation chamber and allowed to sediment over 30 minutes. Images were acquired at 20x magnification. Independent experiments were repeated six times. Both green and red cells were counted manually and normalized to the total number of cells that sedimented during the 30 min period. Both the percentage of cells that sedimented at five-minute intervals and the time required for half the cells to sediment (sedimentation half-time) were determined.

### 2.3 External Magnetic Field Assay

The direct visualization of cell distribution within the large fluid compartments is challenging, and BLI signal dramatically facilitates cell quantitation in these conditions. Thus for visualization and quantification purposes, HUCB-NSCs were transduced with a lentivector encoding the firefly luciferase under a constitutive CMV promoter as previously described [25]. With a transduction efficiency of nearly 100%, further selection was not needed. Cells were labeled with rhodamine-SPIO as described above, and unlabeled cells were used as control. For these experiments, cells were suspended in culture medium at a density of 10^4^ cells/µl. NMR tubes (New Era Enterprises, INC) were filled with 10 mM PBS and placed vertically on a stand. A permanent neodymium magnet with a diameter of 2 inches (Magnet4less, INC) was mounted on a separate stand at the mid-height level of the NMR tube. The distance from the NMR tube to the magnet was adjusted during the experiment, ranging from 0 to 14 cm at 2 cm increments. The external field strength (FS) of the permanent magnet was measured at different distances from the magnet surface using a gaussmeter (GM2, AlphaLab Inc.). With both the NMR tube and the magnet fixed at the desired distance, luciferin (300 ng/ml in 10 mM PBS) was added to the NMR tube, and then 15 µl of 10 mM PBS containing 1.5×10^5^ cells was gently layered on the surface of the 10 mM PBS and allowed to sediment over 15 minutes. After that time, the NMR tube was gently removed from the stand, and placed horizontally in a Xenogen IVIS Spectrum optical imager (Caliper Life Sciences, Billerica, MA) for cell location and quantification measurements. For quantification, the lower half of the NMR tubes was divided into three sections (upper = section A, middle = section B, and lower = section C) and the respective BLI signal was measured for each section depending on the distance from the magnet, and then subjected to regression analysis.

### 2.4 Statistical Analysis

All statistical calculations were performed using PROC MIXED (SAS 9.2). The lowest mean square (LMS) difference test was employed for comparison between means, and the results of the regression analysis were reported as Type III Tests of Fixed Effects, and presented as regression lines.

## Results

### 3.1 Clinical MRI Cell Tracking after ICV Injection

**Figure 1:**
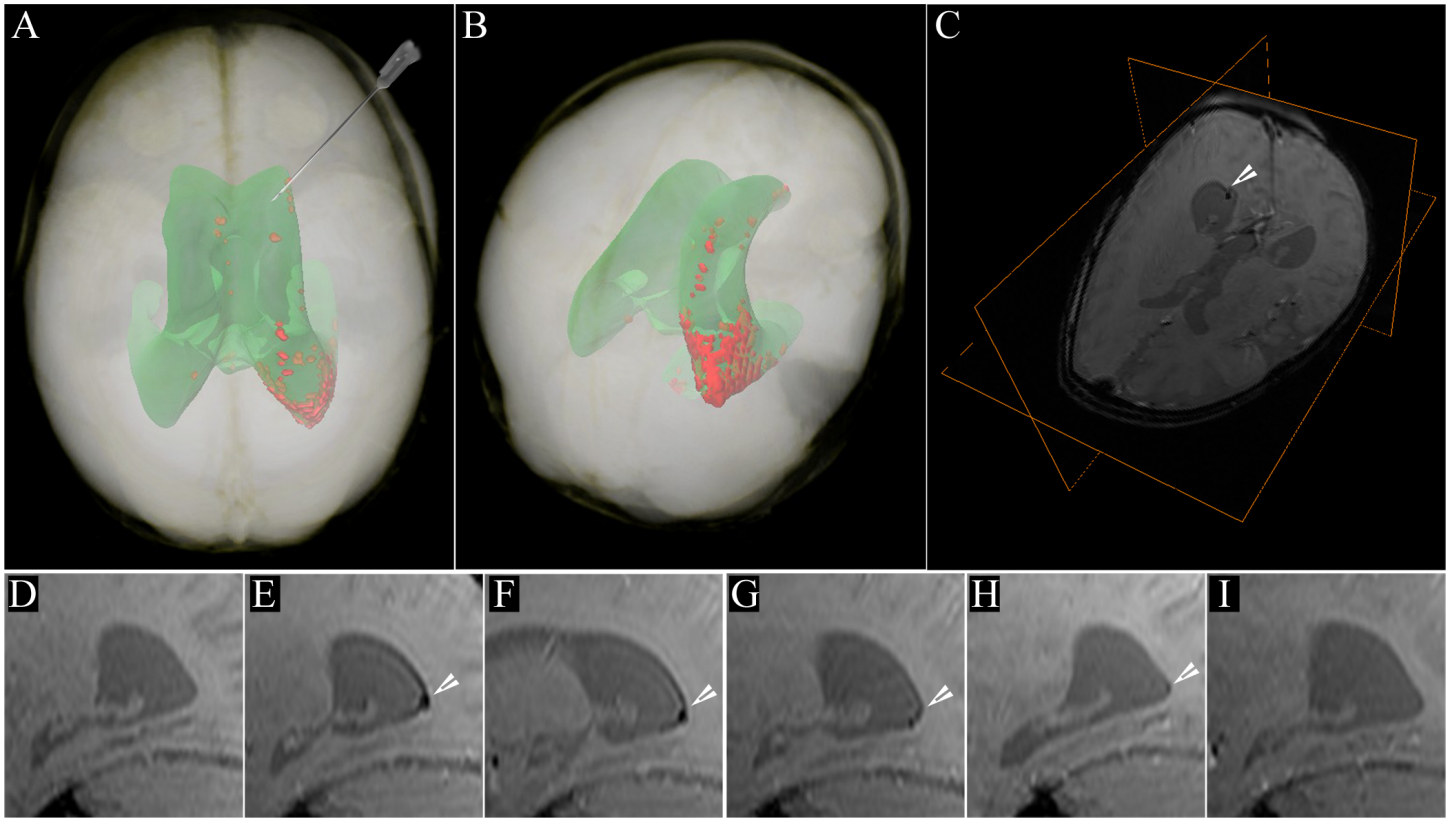
Imaging of SPIO-labeled autologous cord blood derived cells in a patient with global cerebral ischemia. **A**) Volume rendering of MRI data of the patient’s head obtained 24 hours post transplantation. Semi-automatic segmentation is based on pixel intensity, showing the projection of the ventricular system (*green*) and the distribution of the SPIO signal from the transplanted cells within the occipital horn of the right ventricle (*red*). Note the supine configuration of the head, corresponding to positioning during surgery. The route and trajectory of cell transplantation via the frontal horn is represented by the needle. **B**) Postero-superior view of the patient’s head, emphasizing the location of the hypointense SPIO signal from autologous cord blood derived cells transplanted within the occipital horn. **C**) T2*-weighted image with an orthogonal view centered on the cellular SPIO signal in the occipital horn (white arrowhead). **D–I**) Sagittal T2*-weighted MRI scans showing a longitudinal dispersion of SPIO signal within the occipital horn (white arrowheads); **D**) pre-transplantation, **E**) 24 hours post transplantation (PT), **F**) 7 days PT, **G**) 2 months PT, **H**) 4 months PT, and **I**) 33 months PT.

Cell transplantation into the right frontal horn of the lateral ventricle resulted in a focal deposition of cells within the ipsilateral occipital horn, as detected on 24 h post-operative MR scans (Fig. 1 A,B,C, E). Over a period of four months, the cell location within the occipital horn did not change, although a gradual decrease of signal was apparent (Fig. 1 D–H). Very few hypointense regions were observed in other parts of the ventricular system, including the contralateral lateral ventricle or the fourth ventricle. At the long-term follow-up visit (33 months post transplantation), the SPIO-related hypointense signals were no longer detectable (Fig. 1I). MRI at that time did not detect any abnormality that could be attributed to tumor or overgrowth of transplanted cells. There was no major change in the patient’s neurological status over a period of 33 months after transplantation. Despite the poor prognosis, the patient survived without deterioration for an additional three years.

### 3.2 Effect of SPIO-labeling on HUCB-NSC Sedimentation

**Figure 2:**
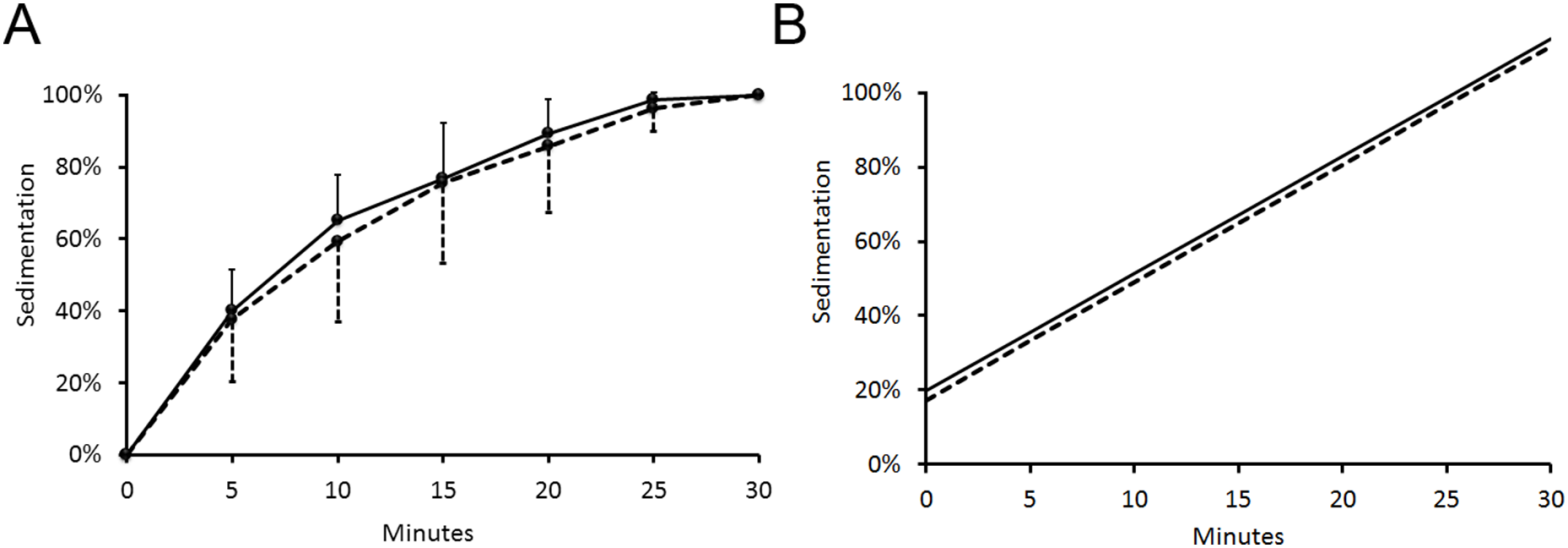
Time course of sedimentation for SPIO-labeled (dashed line) and unlabeled (solid line) HUCB-NSC (n = 6) presented as means±SD (A) and regression lines (B). No statistically significant difference was observed between SPIO-labeled and unlabeled cells.

The number of sedimented cells measured at five-minute intervals did not differ between SPIO-labeled and unlabeled HUCB-NSC at any given time point (p = NS, Fig. 2A). The sedimentation half-time amounted to 7.5±3.3 minutes for unlabeled HUCB-NSC and 9.8±5.9 minutes for cells labeled with SPIO (p = NS). Minute-by-minute evaluation revealed that the majority of cells sedimented to the bottom of the chamber within the first seven minutes. Regression analysis did not also reveal a significant difference in the speed of cell sedimentation between SPIO-labeled HUCB-NSC and cells not labeled with SPIO (Num DF = 1, Den DF = 357, F = 2.33, p = 0.13, Fig. 2B), while, as expected has shown decrease of number of sedimented cells over time, with no further sedimentation observed after 30 minutes (Num DF = 1, Den DF = 357, F = 946.1, p<0.0001).

### 3.3 The Distribution of SPIO-labeled HUCB-NSC can be Guided by Applying an External Magnet

**Figure 3:**
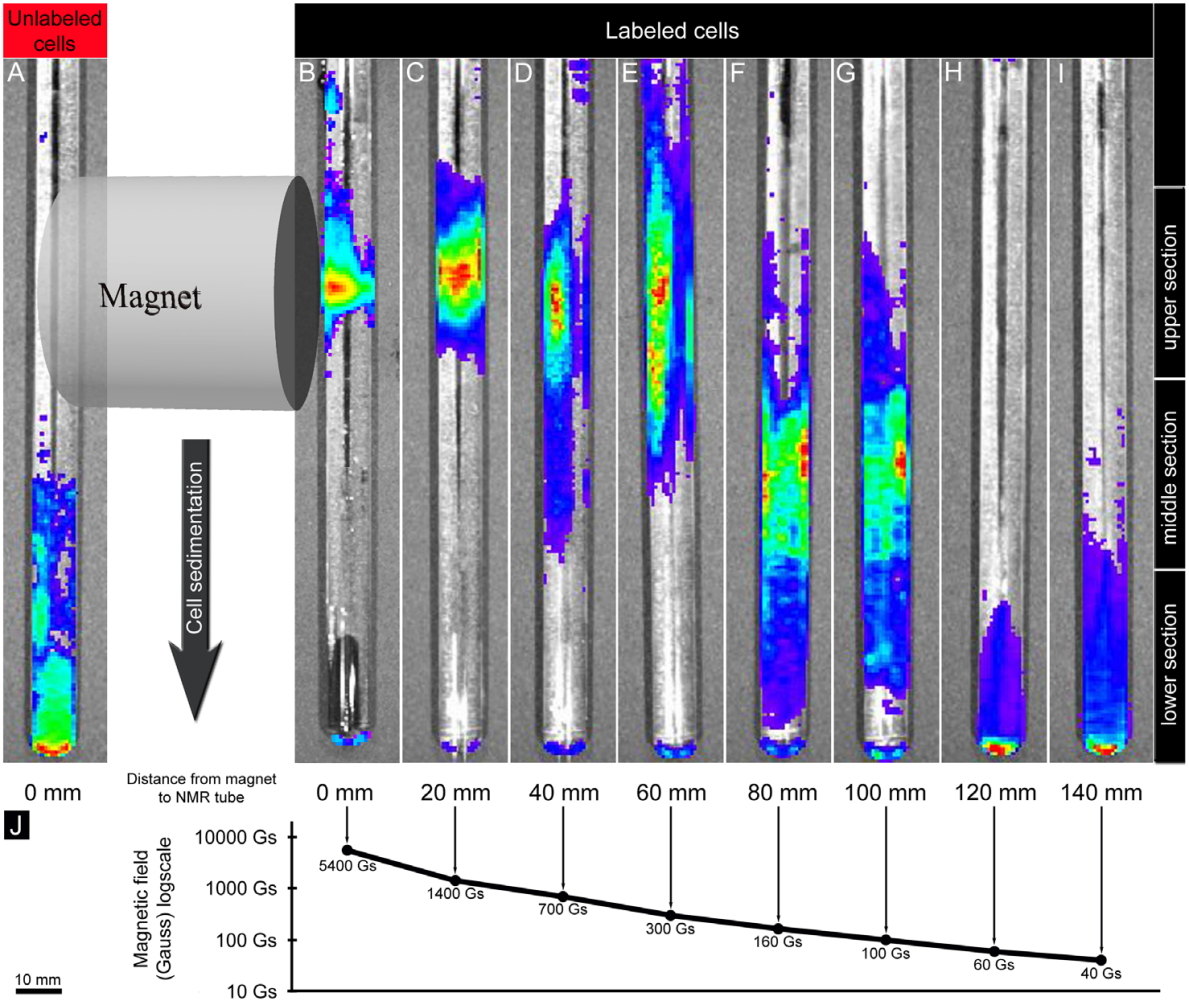
The effect of applying an external magnetic field on HUCB-NSC sedimentation process. Scale bar for the magnet and the tubes is indicated on bottom left.

**Figure 4:**
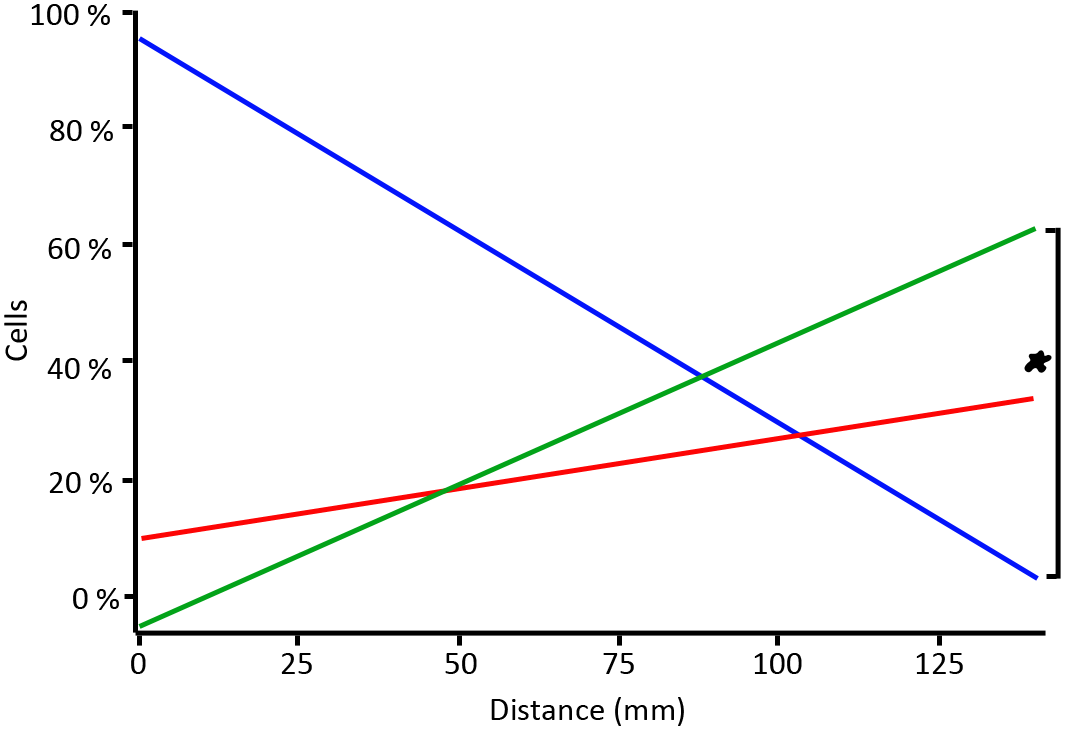
Regression lines show the presence of cells within particular sections in a function of distance between the magnet and the chamber. Statistically significant difference was observed between the cell presence in the upper and lower sections (marked by asterisk). Upper section = green line, middle section = red line, lower section = green line.

We observed that unlabeled HUCB-NSC are not affected by an external magnetic field in terms of sedimentation rate or direction (Fig. 3A). When the magnet was placed directly at the sedimentation tube (FS = 5400 Gs), it enabled us to position the cells exactly at the height corresponding to the center of the magnet (Fig. 3B). When the magnet was placed 2 cm away from the tube containing SPIO-labeled HUCB-NSC (FS = 1400 Gs) it was apparent that most of the cells localized at the magnet level, but were more dispersed (Fig. 3C). Increasing the distance between the tube and the magnet to 4 (FS = 700 Gs) and 6 (FS = 300 Gs) cm still showed a strong pulling effect on labeled cells (Fig. 3D,E). At a distance of 8 (FS = 160 Gs) and 10 (FS = 100 Gs) cm, the effect was reduced, with the cells located between the lower limit of the magnet and the bottom of the tube (Fig. 3F,G), but the force was still sufficient for the cells to prevent sedimentation. At a distance of 12 (FS = 60 Gs) and 14 (FS = 40 Gs) cm the cells sedimented normally (Fig. 3H,I). The strength of magnetic field at each distance was also shown for reference (Fig. 3J). For comparison to the strength of MR scanners the highest used *in vitro* field of 5400 Gs equals 0.54T. Regression analysis confirmed that the distance between the magnet and the chamber had a statistically significant impact on cell location within sections A (upper section) (Num DF = 1, Den DF = 6, F = 17.45, p = 0.0058) and C (lower section) (Num DF = 1, Den DF = 6, F = 6.20, p = 0.0471) (Fig. 4).

## Discussion

Stem cell therapy for neurological disorders is still in its infancy [26]. Nevertheless, it is being offered to patients in several centers located in less-developed countries worldwide [27]–[32]. Such commercial, profit-driven initiatives do not meet the criteria for evidence-based medicine, and this was part of the reason that stem cell transplant services provided by a private clinic in Germany were banned (http://www.xcell-center.com). The International Society for Stem Cell Research recently stepped up efforts to provide guidelines for both patients and clinicians. It is now commonly agreed that the implementation of non-invasive cell tracking techniques is highly desirable, as it could provide a means by which to correlate the presence of transplanted cells with functional improvement. Not including monitoring of cell delivery and subsequent cell distribution in clinical trials has been criticized [33].

This study is one of the first clinical MRI cell tracking trials that aims to demonstrate the feasibility of this approach. This study was particularly focused on the special circumstances associated with an ICV delivery route and the specific benefits of applying MRI cell tracking in this clinical scenario. Due to the absence of focal brain injury, intraparenchymal cell injection was not an option in this patient and alternative routes were considered. The long period from ischemic insult (six months) did not favor an intravascular route, as a potential restoration of the disrupted of the blood-brain barrier (BBB) was expected to occur at this time point. A CSF-mediated route of cell transplantation was considered superior, as it can deliver cells beyond the BBB while still ensuring cell access to a large area of the CNS. CSF is produced mainly in the lateral ventricles by the choroid plexus and flows toward all areas of the ventricular system, emptying within the posterior cranial fossa into the subarachnoid space, and is then finally absorbed into vascular system. In normal physiological conditions, the CSF never returns from the subarachnoid space to the ventricular system. An important implication for cell transplantation is that the ventricular system is lined with an ependyma, with this interface being highly permeable to proteins and passable by cells [34], making the ventricular system an excellent gateway to the brain parenchyma. In contrast, CSF in the subarachnoid space is separated from the brain by the pia mater, a membrane composed of fibrous, impermeable tissue [35]. Thus, lumbar and sub-occipital punctures, although being considered safer procedures, were abandoned in favor of ICV cell delivery. Although access to the ventricles is not entirely non-invasive, it offers an avenue to deliver cells throughout the entire neuroaxis to large areas of the brain.

It has never been reported in humans whether therapeutic cells deposited within the ventricular system are carried along with the CSF flow and distribute throughout the entire neuroaxis. We selected the frontal horns for the site of cell injection, as this is the most commonly used site for ventricular puncture, with a minimal risk for neurosurgical complications. After cell delivery, we could easily identify the hypointense SPIO signal of transplanted cells within the occipital horn, which was the lowest point with the patient in the supine position. Unexpectedly, cells did not distribute throughout the entire brain, but were found to sediment locally in the CSF. Longitudinal imaging over 33 months demonstrated a gradual decrease of signal, with a complete disappearance of the signal at 33 months follow-up. The biological significance of rather short-term retained SPIO signal has yet to be explained. It was shown in rodent studies that neural progenitors transplanted to the lateral ventricles can lose SPIO label before entering the brain parenchyma [34]. In addition, cell death is associated with a longer persistence of SPIO signal as compared to surviving, proliferating cells. There, the persistent hypointense signal was derived from amorphous extracellular iron complexes, presumably released from dying cells [36]. Thus, a gradual decrease of signal after transplantation may indicate cell survival and proliferation or even migration into the brain parenchyma. The detection of widely distributed transplanted cells within the brain parenchyma might be a barrier and since iron oxide provides a very strong signal, probably the limit of MRI has been reached. Due to a very high sensitivity nuclear medicine might be used in future, but no satisfactory PET or SPECT markers for cell detection beyond the blood-brain barrier (BBB) have been found as yet.

The surprising localization of transplanted cells by MRI spurred the *in vitro* experiments geared toward the explanation of observed phenomenon. Although SPIO-labeling has in general no adverse effect on cell function, we minimized any possible side effects by labeling only 20% of transplanted cells. From the MR images, it was apparent that cells rapidly sedimented into the occipital horn. We hypothesized that perhaps SPIO-labeled cells were heavier than unlabeled cells and would sediment with gravity more easily, even against the forces of CSF circulation. Thus, we compared the *in vitro* sedimentation behavior of both SPIO-labeled and unlabeled cells using human umbilical cord blood derived neural stem cells (HUCB-NSC) within a fluid compartment, and found no difference between the two cell populations, both reaching the bottom of a 15 cm-high chamber within one minute. We therefore conclude that, for future studies, only a small subset of transplanted cells can be labeled in order to provide adequate information about the distribution of the entire cell population. The first clinical MRI cell tracking using cancer vaccines also used partial SPIO-labeling, i.e. 50% of all cells [37].

As we have shown here with an *in vitro* assay, directing the position of SPIO-labeled HUCB-NSC against the force of gravity is feasible with the use of an external magnetic field. Even a readily available, inexpensive neodymium magnet is sufficient to remotely direct the cells over a distance compatible with the size of the lateral ventricles in the human brain. It may be possible that applying an external magnetic field may be used to direct cells within the ventricular fluid compartment toward the desired part of the ventricular wall located closest to the focal lesion. A stable pattern of the exact location of hypointensities in our patient over time suggests that, once cells adhere to the ependyma, they do not redistribute. As a consequence, any manipulation of cell distribution should be performed at the time of cell injection, with a small window of opportunity of about 10 minutes following transplantation. This very narrow time frame results from observation that cell sedimentation is a rapid process, which is completed in a few minutes.

The observation that SPIO-labeled HUCB-NSC are strongly attracted by an external magnetic field gives rise to the question if the high magnetic field generated by the MR scanner may also have contributed to the observed local cellular distribution. Nonetheless, in case of the patient, the cellular location did not change over time between any of the multiple MR imaging sessions. In addition, with cord blood derived cells deposited within the occipital horn, the magnetic field of a 1.5 T MR scanner would pull the cells rostrally toward the body of the lateral ventricle, which was not observed, as the cells remained within the occipital horn. Thus, it appears highly unlikely that the MR imaging procedure itself had any effect on the distribution of SPIO-labeled cord blood derived stem cells.

In conclusion, we have shown that MRI cell tracking is a powerful clinical tool to assess the distribution of transplanted cells in vivo. Following transplantation to the frontal horn, autologous cord blood derived cells unexpectedly remained localized within a well-defined region of the ependyma in the occipital horn for at least 4 months post transplantation. An external magnet may possibly be used to direct SPIO-labeled cells within a fluid compartment such as the ventricular system. For future implementation, MRI cell tracking will be mandatory to evaluate the effectiveness and outcome of this approach.
